# VITAMIN D DEFICIENCY AND FASTER EPIGENETIC AGING: RESULTS FROM THE BERLIN AGING STUDY II (BASE-II)

**DOI:** 10.1093/geroni/igac059.1738

**Published:** 2022-12-20

**Authors:** Valentin Vetter, Yasmine Sommerer, Christian Kalies, Dominik Spira, Lars Bertram, Ilja Demuth

**Affiliations:** Charité - Universitätsmedizin Berlin, Berlin, Berlin, Germany; University of Lübeck, Lübeck, Schleswig-Holstein, Germany; Charité – Universitätsmedizin Berlin, Berlin, Berlin, Germany; Charité - Universitätsmedizin Berlin, Berlin, Berlin, Germany; University of Lübeck, Lübeck, Schleswig-Holstein, Germany; Charité Universitätsmedizin Berlin, Berlin, Berlin, Germany

## Abstract

Particularly older people are at risk for deficient vitamin D levels, as their capability for cutaneous synthetization is lower and they are often less exposed to sunlight. However, the resulting impact on one individual’s health is not fully understood. To examine potential consequences of low vitamin D levels for health of older people, we examined its relationship with a DNA related biomarker of aging, DNA methylation age acceleration (DNAmAA). Several epigenetic clocks, that are used to estimate this parameter from methylation fractions of specific cytosine-phosphate-guanine sites, are available and differ in aspects of aging they represent best. Five clocks, 7-CpG clock, Horvath’s clock, Hannum’s clock, PhenoAge, and GrimAge, were available in the longitudinal BASE-II cohort (n=1,100) at follow-up examination. DNAmAA estimated from 7-CpG clock and GrimAge was associated with vitamin D levels in covariate adjusted regression analysis. Additionally, a quasi-interventional study design was employed and showed 2.6 year lower 7-CpG DNAmAA (p=0.011) and 1.3-year lower Horvath DNAmAA (p=0.042) in vitamin D deficient participants that chose to start vitamin D supplementation during the follow-up period of 7.4 ± 1.5 years compared to a matched control-group of participants with untreated vitamin D deficiency. No statistically significant difference was found between sufficiently treated participants with vitamin D deficiency and a matched group of participants that reached sufficient vitamin D level without supplementation. Although validation of our results in a randomized controlled trial is needed, our findings suggest, that vitamin D deficiency associated higher rates of epigenetic aging can potentially be reversed through supplementation.

